# Reply: Questionable recommendation for LPS for IVF/ICSI in ESHRE guideline 2019: ovarian stimulation for IVF/ICSI

**DOI:** 10.1093/hropen/hoab006

**Published:** 2021-03-03

**Authors:** Frank Broekmans, Peter Humaidan, George Lainas, Mira Töyli, Nathalie Le Clef, Nathalie Vermeulen

**Affiliations:** 1 Department of Reproductive Medicine and Gynecology, University Medical Center Utrecht, The Netherlands; 2 The Fertility Clinic, Skive Regional Hospital, Faculty of Health, Aarhus University, Skive, Denmark; 3 Eugonia Assisted Reproduction Unit, 7 Ventiri Street, 11528 Athens, Greece; 4 Fertinova Clinics, Helsinki, Finland; 5 European Society of Human Reproduction and Embryology, Belgium

Sir,

We have read with interest the concerns from Dr. Piette (2021), consultant for Besins Healthcare, regarding the recommendation on dydrogesterone in the ESHRE Guideline on ovarian stimulation.

We were aware of the error in the recommendation regarding progesterone for luteal phase support prior to the correspondence with Dr. Piette was received, and steps were already taken to correct this. The error was introduced by the publisher during typesetting. The error has been corrected and the paper has been republished with an accompanying erratum.

In line with the procedures for the development of evidence-based guidelines (https://www.eshre.eu/Guidelines-and-Legal/Guidelines/Guideline-development-process), the guideline group performed a formal literature search on efficacy and safety of dydrogesterone for luteal phase support. Studies reporting on safety of dydrogesterone for luteal support in IVF (Lotus I and II; Tournaye, 2017; Griesinger, 2018) were considered, but we also searched for (more long-term) safety data which are only available in the context of prevention of first trimester miscarriage (Carp, 2012; Nadarajah *et al*., 2017; Saccone *et al*., 2017; Zaqout *et al*., 2015).

After reviewing the available evidence on efficacy and safety in IVF, the guideline development group agreed to formulate a conditional recommendation in favor of dydrogesterone using the phrase “probably recommended” in line with the GRADE/DECIDE system. The recommendation was formulated as “Dydrogesterone is probably recommended for luteal phase support.”

To ensure readability, the HROpen paper is limited to the list of all recommendations, with a clear reference to the full guideline and additional material on the ESHRE website for further information and resources. The full guideline provides a description of the evidence and considerations that substantiated this recommendation. We do not consider it misleading that the summary paper published in HROpen includes only the recommendations.

As part of the guideline development protocol, the guideline draft was open for review for 6 weeks, between 12 February and 26 March 2019. All reviewers, their comments and the reply of the guideline development group are summarized in the review report, which is published on the ESHRE website as supporting documentation to the guideline (https://www.eshre.eu/OSguide).

The opportunity for people outside the guideline group to formulate comments and concerns, and the transparency in which these are either addressed or refuted, is an important process in guideline development ensuring trustworthy and independent guidelines. However, adapting recommendations based on comments formulated outside the stakeholder process may jeopardize the objectivity of the guideline.

Of course, if new evidence is published, recommendations may need to be revised. As such, we have performed an update of the literature published on dydrogesterone for luteal support in IVF which confirmed the lack of new data published in this context. The meta-analysis by Koren *et al*. (2020) referenced by Dr. Piette, does not focus on luteal phase support, but on prevention of first trimester miscarriage. Furthermore, the conclusion of the meta-analysis regarding dydrogesterone, is mainly based on one large dataset, which was published as a conference abstract, by the same author conducting the meta-analysis (Koren *et al*., 2020). In the context of providing recommendations for clinical practice, such data would be considered invalid, at least until they have been published in a peer-reviewed journal and the quality of the data can be assessed. The scheduled update of the guideline is planned in 3 years.

In conclusion, and although we value any feedback to the ESHRE Guidelines, the guideline group has no arguments to consider an adaptation of its current recommendation regarding dydrogesterone for luteal support in IVF/ICSI.
